# Vaginal Microbiomes Associated With Aerobic Vaginitis and Bacterial Vaginosis

**DOI:** 10.3389/fpubh.2018.00078

**Published:** 2018-03-26

**Authors:** Evelyn Kaambo, Charlene Africa, Ramadhani Chambuso, Jo-Ann Shelley Passmore

**Affiliations:** ^1^Genital Mucosal HIV and STI Research Group, Division of Medical Virology, Department of Pathology, Faculty of Health Sciences, University of Cape Town, Medical Virology Laboratory, Cape Town, South Africa; ^2^Genital Mucosal HIV and STI Research Group, Department of Pathology, Division of Medical Virology, NRF-DST CAPRISA CENTRE OF EXCELENCE, University of Cape Town, Cape Town, South Africa; ^3^Department of Biochemistry and Medical Microbiology, University of Namibia Medical School, Windhoek, Namibia; ^4^MENIS, Medical Biosciences, University of the Western Cape, Bellville, South Africa; ^5^Division of Medical Virology, Department of Pathology, University of Cape Town, Cape Town, South Africa; ^6^Department of Gynecology, Morogoro Regional Referral Hospital, Morogoro, Tanzania; ^7^National Health Laboratory Service, Johannesburg, South Africa

**Keywords:** aerobic vaginitis, bacterial vaginosis, vaginal microbiomes, group B *Streptococcus*, preterm birth

## Abstract

A healthy vaginal microbiota is considered to be significant for maintaining vaginal health and preventing infections. However, certain vaginal bacterial commensal species serve an important first line of defense of the body. Any disruption of this microbial barrier might result in a number of urogenital conditions including aerobic vaginitis (AV) and bacterial vaginosis (BV). The health of the vagina is closely associated with inhabitant microbiota. Furthermore, these microbes maintain a low vaginal pH, prevent the acquisition of pathogens, stimulate or moderate the local innate immune system, and further protect against complications during pregnancies. Therefore, this review will focus on vaginal microbial “health” in the lower reproductive tract of women and on the physiological characteristics that determine the well-being of reproductive health. In addition, we explore the distinct versus shared characteristics of BV and AV, which are commonly associated with increased risk for preterm delivery.

## Introduction

Commensal microbiota associated with the human body influence many aspects of fetal development, physiological function, immunity at mucosal surfaces, susceptibility to diseases and ability to assimilate nutrients (1). As such, commensals in the lower female reproductive tract are significant in maintaining vaginal health as well preventing infections (2).

Human vaginal infections are associated with significantly increased risk of preterm birth in women (3, 4). If untreated, they can lead to pelvic inflammatory diseases (PID), which can cause tubal infertility, ectopic pregnancy, reproductive dysfunction, and adverse pregnancy outcomes (including preterm delivery and low birth weight) (3, 4). Vaginal infections may contribute to the progression of cervical dysplasia, increased risk of post-delivery infections, HIV, and herpes simplex virus-2 (HSV-2) acquisition and transmission (3, 5, 6). However, the unexpected pregnancy outcome and premature birth due to aerobic vaginitis (AV) and bacterial vaginosis (BV) infections in prenatal health services among asymptomatic pregnant women is high in Africa and worldwide (7).

The contribution of AV and BV to vaginal health and pregnancy outcome has been investigated for over a century, yet they remain incompletely understood (8). For example, in epidemiologic studies, it has been suggested that having multiple sexual partners, increased maternal age, previous spontaneous abortions, and altered vaginal bacterial communities (including decreased *Lactobacillus* species and concurrent colonization with *Candida* species) are the risk factors for vaginal colonization with microbes associated with endogenous infection such as AV and BV (8, 9). However, the unexpected pregnancy outcome and premature birth due to AV and BV infections in prenatal health services among asymptomatic pregnant women are high in Africa and worldwide (7).

## The Healthy Vaginal Microbiome

The vagina is a highly nutrient-rich chamber for microbes (10). As a result, the composition of the vaginal microbiota is affected by numerous host factors, including age, changes in hormone levels (during the menstrual cycle, during menopause, pregnancy, or as a result of hormone contraceptive use), other genital infections, as well as sexual and hygiene practices (1, 11). Most of these commensal microbiotas do exist in mutualistic relationships with their human hosts, although a few are opportunistic pathogens that can cause chronic infections, preterm delivery, or life-threatening maternal and fetal diseases (1, 6).

## Mutualistic Host–Commensal Relationships in the Lower Female Reproductive Tract

Several bacterial species colonize both the gastrointestinal and reproductive tracts of women, in addition, the rectum has been suggested to provide an essential source of organisms that commonly colonize the vagina (11). This is of significance since bacteria are repeatedly shed from the body in vaginal secretions, and bacterial regrowth must happen to replenish their number (1).

Some of the required nutrients are derived from host epithelial dead cells, while others are derived from glandular secretions in the lower reproductive tract (1). The vaginal epithelium has a limited blood supply and depends on diffusion of glucose, oxygen, and numerous vital nutrients from underlying submucosal tissues that result in the relatively anaerobic environment at the genital mucosa (12). In addition, the vaginal mucosa undergoes recurring cycles of proliferation of the basal layer, maturation, and desquamation into the vaginal lumen (12).

The vagina harbors a collection of microbes that are distinct from other human surfaces and mucosal sites, with a reduced microbial diversity dominated by *Lactobacillus* species, believed to cause acidification of this environment (13). Furthermore, the ability of lactobacilli to colonize the vaginal mucosa is influenced by the route of delivery and extent of adhesion to vaginal epithelial cells (14).

## Physiological Vaginal Discharge and the Microbiome

Notable changes occur in the physiology, immunology and microbiology of the female reproductive tract following the onset of puberty. The lower reproductive tract before puberty appears red, due to many blood vessels under the thin hypo-estrogenic mucosa (15). Before puberty, the vaginal pH is close to neutral but not <0.4.7, and the microbiome is closely related to the predominant fecal bacterial species.

Healthy women of reproductive age typically have some degree of vaginal discharge, with the quantity and type of secretion varying during the menstrual cycle as a result of hormonal fluctuations (15). Before ovulation, estrogen levels increase, altering cervical mucus from a thicker, sticky consistency to clearer, wetter, more elastic and slippery in preparation for fertilization (16). After ovulation, estrogen levels decline and progesterone levels increase, resulting in cervical mucus becoming thicker, sticky, and less permissive to sperm motility (17).

With the increase of the estrogen levels around puberty, the genital mucosa thickens, becoming a lighter pink in color and becomes colonized with *Lactobacillus* species which produce lactic acid and hydrogen peroxide (H_2_O_2_) to lower the pH below 4.5 (18). Production of H_2_O_2_ by certain *Lactobacillus* species is thought to be essential in inhibiting the overgrowth of normal facultative anaerobes (15, 19).

Following puberty, adolescent genital secretions are produced by a number of glands, including the vulva, sebaceous and sweat, appearing white (15). In addition to mucus, other common constituents of these secretions include exfoliated epithelial cells, secretions from the upper reproductive tract, immune molecules, and metabolic by-products (15). This white discharge characteristically does not adhere to the vaginal walls, pools in the posterior fornix, and has an acidic pH of <4.5 (16). In adult women, normal variations in the menstrual cycle, hormonal contraception use, and different stressors may alter the consistency, color, and amount of vaginal discharge (18).

Due to increasing estrogen levels following puberty, sexually matured women have a lower reproductive tract that favors lactobacilli colonization. Estrogen results in thicker stratified epithelia and higher glycogen concentrations with *Lactobacillus* species contributing to a lower pH to ≤4.5 in the vagina (10), due to lactic acid and H_2_O_2_ production. In addition to *Lactobacillus* species, some commensal microorganisms commonly identified in the lower reproductive tract include *Candida albicans, Staphylococcus aureus*, and *Streptococcus agalactiae* (group B *Streptococcus*) (20). These can cause changes in discharge if they are not controlled and become overgrown (20).

## Other Common Causes of Abnormal Vaginal Discharge

Abnormal vaginal discharge differs in color and consistency (thin, thick, frothy, yellow, green, gray, or white) compared with physiological (normal) discharge, and frequently is associated with other symptoms, including itching, fishy, and foul smelling (15). Abnormal discharge is typically caused by vaginal infections, although some relatively common non-infectious etiologies occur as well (21).

Infectious causes of abnormal discharge usually include sexually transmitted infections including *Chlamydia trachomatis, Neisseria gonorrhoeae, Trichomonas vaginalis, Ureaplasma*, and less commonly HSV-2 and endogenous infections (i.e. dysbiotic outgrowths of common commensal bacterial such as in AV and BV).

Non-infectious causes of vaginal discharge include vaginal douching, using perfumed soaps for cleaning, some laundry detergents, toilet paper, fabric softeners, and other feminine hygiene products that may act as irritants and cause abnormal discharge (22). Sometimes, foreign bodies (such as a retained tampon, condom, or toilet papers) may result in a malodorous or bloody discharge (22).

## Bacterial Vaginosis

Bacterial vaginosis is defined according to the presence of clinical symptoms and increased vaginal pH, typically ≥4.5, existence of white adherent discharge that contains exfoliated epithelial cells with Gram-variable polymorphic rod-shaped bacteria attached to their surfaces (clue cells), and a fishy odor (23). BV is typically polymicrobial, characterized by the presence of mainly anaerobic microorganisms including *Gardnerella vaginalis, Prevotella* species, and *Mycoplasma hominis, Mobiluncus* species (8, 24, 25). An increased risk of PID, STIs (in addition to HIV infection), and preterm delivery in pregnant women is associated with BV (13, 26).

## Aerobic Vaginitis

Aerobic vaginitis was first characterized in 2002 (21), as a vaginal condition distinct from BV, which may require different clinical management and have distinct clinical risks (27). Like BV, AV is defined by disruption in *Lactobacillus* dominance but is accompanied by more extreme inflammatory changes than BV and the presence of mainly aerobic enteric commensals or pathogens, including Group B *Streptococcus* (*S. agalactiae*), *Enterococcus faecalis, Escherichia coli*, and *S. aureus* (20, 27–31). AV has been observed in 8–11% of pregnant women (27, 28) and in 5–24% of women reporting vaginal complaints (24, 27).

In certain cases, AV is associated with more genital inflammation, increased numbers of leukocytes visible in vaginal smears, with increased activity to pathogens [termed “toxic leukocytes” (32)]. Women with AV tend to have thinner vaginal mucosa than those with BV, with increased numbers of intermediate and parabasal cells in vaginal smears, indicative of increased turnover and desquamation of superficial epithelial cell layers (32). The comparison of clinical and microbiological characteristics of AV and BV is summarized in Table 1.

**Table 1:** Comparison of clinical and microbiological characteristics of aerobic vaginitis (AV) and bacterial vaginosis (BV).

Characteristics	AV	BV
Clinical		
pH	>4.5, usually >6	≥4.5
Discharge	Yellowish	White, homogenous
Epithelial inflammation	Present	None
Shed epithelial cells	Parabasal cells	Clue cells
Whiff test (fishy amine odor)	Negative	Positive
Lactobacilli species	Displaced	Displaced

Microbiological	Group B *Streptococcus*	*Gardnerella vaginalis*
Common pathogens	*Enterococcus faecalis*	*Atopobium vaginae*
	*Escherichia coli*	*BVAB2*
	*Staphylococcus aureus*	*Megasphaera* species

Immunological		
Inflammatory cytokines	High	Moderate
Immune reaction (cytokines)	Reactive	None reactive

Treatment	Kanamycin	Metronidazole
	Clindamycin topical	Clindamycin
	Fluoroquinolones	
	i.e., Ciprofloxacin and ofloxacin	

Both BV and AV diagnosis is based on wet mount microscopy, with diagnosis based on deficiency of *Lactobacillus* species (33). Unlike BV, AV wet mounts are positive for cocci or coarse bacilli, positive for parabasal epithelial cells, and positive for vaginal leukocytes (plus their granular aspect) (30). The diagnosis of AV is also based on molecular diagnostic methods and microscopic criteria graded on a quantitative scale (27, 30) (Table 2). Donders et al. (27) have proposed an algorithm for grading AV that is similar to the Nugent scoring system, where the number of points establishes the composite AV score, with the maximum score being 10. Like the Nugent scoring system used for grading BV, the AV score may indicate normal, intermediate, or severe AV (27).

**Table 2:** Microscopic diagnosis criteria for aerobic vaginitis (AV) (27).

AV score	# Lactobacillary grades	Number of leukocytes	Proportion of toxic leukocytes	Background flora	Proportion of parabasal epitheliocytes
0	I and IIa	10/hpf^a^	None or sporadic	Unremarkable or cytolysis	None or <1%
1	IIb	>10/hpf^a^ and ≤10/Epithelial cell	50% of Leukocytes	Small coliform bacilli	≤10%
2	III	10/Epithelial cell	50% of Leukocytes	Cocci or chains	>10%

*^a^High-power field (hpf) (400× magnification)*.

*# Lactobacillary grade I consists of numerous pleomorphic lactobacilli and no other bacteria*.

*# Lactobacillary grade IIa consists of Lactobacillus species predominance but with some mixed microbiota: LBG IIb consists also of a mixed microbiota but the proportion of lactobacilli being severely decreased as a result of an increased number of the bacteria*.

*# Lactobacillary grade III consists of lactobacilli severely depressed or absent because of the overgrowth of other bacteria*.

Lactobacillary grades are the basis for a composite score to which the following four variables have been added: (a) proportional number of lactobacilli; (b) the presence of toxic leukocytes; (c) the presence of parabasal epithelial cells; and (d) the type of background microbiota (27).

Aerobic vaginitis, without an accurate diagnosis, might be incorrectly diagnosed as BV, leading to incorrect treatment or even more severe complications of AV such as desquamative inflammatory vaginitis, which is considered to increase the risk of preterm delivery, chorioamnionitis, and funisitis of the fetus during pregnancy (7).

## *S. agalactiae* in AV

*Streptococcus agalactiae* (Group B *Streptococcus*) is a member of the commensal microbiota of the human intestinal and genitourinary tracts and is also an important pathogen in AV and other human infections (34). Colonization with GBS is the major risk factor for early-onset invasive GBS disease in newborns (35, 36). GBS rarely cause infections in healthy adults; however, occasionally it may cause morbidity in the elderly, in pregnant women, and in patients with underlying predisposing conditions (34, 35).

Maternal colonization with GBS in pregnant women at delivery is associated with neonatal sepsis, meningitis, and pneumonia (7, 13). The composition of the vaginal microbiota is considered to be very different from that present at other human body sites (13). Currently, GBS is considered to be the leading cause of early-onset sepsis worldwide (37, 38). In addition, GBS and *E. faecalis* are associated with preterm birth, very low birth weight delivery, and puerperal sepsis, which cause substantial morbidity and mortality in sub-Saharan Africa (4, 37, 39).

Prevention of GBS infections in pregnant women and their infants through maternal immunization has been a vision for decades and now is an achievable goal (40). Although GBS can cause asymptomatic bacteriuria, lower urinary tract infection, and acute pyelonephritis, most pregnant women have asymptomatic bacteriuria (40–42). Intrauterine fetal death, chorioamnionitis, and early-onset neonatal disease all are increased among women with untreated GBS bacteriuria (40, 43).

## Treatment for AV and BV

There is no generally accepted clinical strategy for treating AV (44). AV is treated with antibiotics with intrinsic activity against bacteria of fecal origin, in addition to ensuring minimal interference with vaginal *Lactobacillus* species (44). Antibiotics alone may not be adequate for most patients with AV due to the amount of inflammation typically associated with this condition, including infiltrating leukocytes and parabasal cells (27).

For this reason, the appropriate diagnosis and distinction between AV and BV is very crucial as their treatment is different. Unlike BV, AV does not respond well to metronidazole, which is commonly used for the treatment of *T. vaginalis* and BV (33). Clindamycin is therefore considered to be a better choice than metronidazole for pregnant women with an abnormal vaginal microbiota (45). Fluoroquinolones, such as ciprofloxacin and ofloxacin, have been used in treatment because they have little effect on the normal flora allowing for a rapid recovery from AV (45).

Han et al. (44), proposed that best practice for treating AV would be to base it on microscopy findings with local treatment with any combination of antibiotics (for the infectious agent), topical steroids (to reduce inflammation), and estrogen (to treat atrophy). To accomplish a rapid as well as short-term improvement for severe symptoms, systemic therapy with moxifloxacin can be used, especially in infections with group B streptococci and for methicillin resistant *S. aureus* (4).

In sub-Saharan Africa countries, sophisticated microbiota detection and administration of clinical interventions are definitely impossible at the moment. Assistance in lowering the morbidity and mortality rate is needed and can only occur if scientists in developing countries use the latest molecular technologies as well genetic investigations to have better understanding of the causes of preterm labor, particularly the triggers produced directly by bacteria or *via* the host, that can lead to irreversible preterm reactions (31). Furthermore, for diagnosis and interventional purpose, products to be applied to the developing world, as well remote and rural areas, must be simple, easy to use, and well understood.

Direct use of probiotics inserted into the vagina is worthy of consideration. However, retention of accessibility of the strains, which are available to the participants in need, appropriate storage, and cost of such capsules, are challenges that will have to be overcome.

## Conclusion

Maintaining a healthy vaginal environment is considered a challenge worldwide. To prevent the risk of infection, health-care professionals should consider enhancing education to improve women’s knowledge of reproductive health and the distinction between AV and BV. We believe that the assessment of healthy vaginal microbiomes, in combination with host genetics, particularly to AV and BV infections and preterm delivery, is urgently needed.

Considering the existing knowledge gap regarding AV, primarily in Africa, there is a need to identify antibiotic sensitivity profiles of the aerobic and anaerobic vaginal pathogens in women of reproductive age to prevent adverse pregnancy outcomes due to opportunistic pathogens, many of which demonstrate antimicrobial resistance (46). The study of vaginal microbiomes will provide an opportunity to define vaginal health, and the link between vaginal infections and commensal opportunistic microbial pathogens in pregnant women. Such surveillance studies will contribute significantly to the literature by establishing epidemiological trends of AV and BV and may well inform about the importance of maternal reproductive health for positive pregnancy outcomes.

## Author Contributions

Each of the following authors contributed equally to this manuscript: EK, CA, RC, and J-AP.

## Conflict of Interest Statement

The authors declare that the research was conducted in the absence of any commercial or financial relationships that could be construed as a potential conflict of interest.

## References

[1] Ma B Forney LJ Ravel J. Vaginal microbiome: rethinking health and disease. Annu Rev Microbiol (2012) 66:371–89. 10.1146/annurev-micro-092611-150157 22746335 PMC3780402

[2] Stojanovic N Plecas D Plesinac S. Normal vaginal flora, disorders and application of probiotics in pregnancy. Arch Gynecol Obstet (2012) 286(2):325–32. 10.1007/s00404-012-2293-7 22437191

[3] Mulu W Yimer M Zenebe Y Abera B. Common causes of vaginal infections and antibiotic susceptibility of aerobic bacterial isolates in women of reproductive age attending at Felegehiwot Referral Hospital, Ethiopia: a cross sectional study. BMC Womens Health (2015) 15:42. 10.1186/s12905-015-0197-y 25968607 PMC4438529

[4] Kaambo E Africa CW The threat of aerobic vaginitis to pregnancy and neonatal morbidity. Afr J Reprod Health (2017) 21(2):109–18. 10.29063/ajrh2017/v21i2.12 29624945

[5] Lamichhane P Joshi D Subedi Y Thapa R Acharya G Lamsal A Study on types of vaginitis and association between bacterial vaginosis and urinary tract infection in pregnant women. Int J Biomed Adv Res (2014) 5(6):305–7. 10.7439/ijbar.v5i6.762

[6] Ravel J Gajer P Abdo Z Schneider GM Koenig SS McCulle SL Vaginal microbiome of reproductive-age women. Proc Natl Acad Sci U S A (2011) 108(Suppl 1):4680–7. 10.1073/pnas.1002611107 20534435 PMC3063603

[7] Krauss-Silva L Almada-Horta A Alves MB Camacho KG Moreira MEL Braga A. Basic vaginal pH, bacterial vaginosis and aerobic vaginitis: prevalence in early pregnancy and risk of spontaneous preterm delivery, a prospective study in a low socioeconomic and multiethnic South American population. BMC Pregnancy Childbirth (2014) 14(1):107. 10.1186/1471-2393-14-107 24641730 PMC3994593

[8] Rampersaud R Randis TM Ratner AJ, editors. Microbiota of the upper and lower genital tract. Semin Fetal Neonatal Med (2012) 17(1):51–7. 10.1016/j.siny.2011.08.006 21920833 PMC3242913

[9] Nardis C Mosca L Mastromarino P. Vaginal microbiota and viral sexually transmitted diseases. Ann Ig (2013) 25(5):443–56. 10.7416/ai.2013.1946 24048183

[10] Danielsson D Teigen PK Moi H. The genital econiche: focus on microbiota and bacterial vaginosis. Ann N Y Acad Sci (2011) 1230(1):48–58. 10.1111/j.1749-6632.2011.06041.x 21824165

[11] El Aila NA Tency I Claeys G Saerens B De Backer E Temmerman M Genotyping of *Streptococcus agalactiae* (group B streptococci) isolated from vaginal and rectal swabs of women at 35–37 weeks of pregnancy. BMC Infect Dis (2009) 9(1):153. 10.1186/1471-2334-9-153 19747377 PMC2753344

[12] Linhares IM Summers PR Larsen B Giraldo PC Witkin SS. Contemporary perspectives on vaginal pH and lactobacilli. Am J Obstet Gynecol (2011) 204(2):120.e1–5. 10.1016/j.ajog.2010.07.010 20832044

[13] Fredricks DN. Molecular methods to describe the spectrum and dynamics of the vaginal microbiota. Anaerobe (2011) 17(4):191–5. 10.1016/j.anaerobe.2011.01.001 21376827 PMC3126881

[14] Barrons R Tassone D. Use of *Lactobacillus* probiotics for bacterial genitourinary infections in women: a review. Clin Ther (2008) 30(3):453–68. 10.1016/j.clinthera.2008.03.013 18405785

[15] Syed TS Braverman PK. Vaginitis in adolescents. Adolesc Med Clin (2004) 15(2):235–51. 10.1016/j.admecli.2004.02.003 15449843

[16] Ilkit M Guzel AB. The epidemiology, pathogenesis, and diagnosis of vulvovaginal candidosis: a mycological perspective. Crit Rev Microbiol (2011) 37(3):250–61. 10.3109/1040841X.2011.576332 21599498

[17] Hickey DK Patel MV Fahey JV Wira CR. Innate and adaptive immunity at mucosal surfaces of the female reproductive tract: stratification and integration of immune protection against the transmission of sexually transmitted infections. J Reprod Immunol (2011) 88(2):185–94. 10.1016/j.jri.2011.01.005 21353708 PMC3094911

[18] Carr PL Felsenstein D Friedman RH. Evaluation and management of vaginitis. J Gen Intern Med (1998) 13(5):335–46. 10.1046/j.1525-1497.1998.00101.x 9613891 PMC1496957

[19] Nyirjesy P. Vaginitis in the adolescent patient. Pediatr Clin North Am (1999) 46(4):733–45. 10.1016/S0031-3955(05)70149-8 10494254

[20] Sangeetha KT Golia S Vasudha CL A study of aerobic bacterial pathogens associated with vaginitis in reproductive age group women (15–45 years) and their sensitivity pattern. Int J Res Med Sci. (2015) 3(9):2268–73. 10.18203/2320-6012.ijrms20150615

[21] Donders GG Vereecken A Bosmans E Dekeersmaecker A Salembier G Spitz B. Definition of a type of abnormal vaginal flora that is distinct from bacterial vaginosis: aerobic vaginitis. BJOG (2002) 109(1):34–43. 10.1111/j.1471-0528.2002.00432.x 11845812

[22] Le Doare K Heath PT. An overview of global GBS epidemiology. Vaccine (2013) 31:D7–12. 10.1016/j.vaccine.2013.01.009 23973349

[23] Turovskiy Y Noll KS Chikindas ML The etiology of bacterial vaginosis. J Appl Microbiol (2011) 110(5):1105–28. 10.1111/j.1365-2672.2011.04977.x 21332897 PMC3072448

[24] Zarbo G Coco L Leanza V Genovese F Leanza G D’Agati A Aerobic vaginitis during pregnancy. Res Obstet Gynecol (2013) 2(2):7–11. 10.5923/j.rog.20130202.01

[25] Srinivasan U Misra D Marazita ML Foxman B. Vaginal and oral microbes, host genotype and preterm birth. Med Hypotheses (2009) 73(6):963–75. 10.1016/j.mehy.2009.06.017 19942083 PMC4026093

[26] Tomusiak A Heczko PB Janeczko J Adamski P Pilarczyk-Zurek M Strus M. Bacterial infections of the lower genital tract in fertile and infertile women from the southeastern Poland. Ginekol Pol (2013) 84(5):352–8. 23819400 10.17772/gp/1588

[27] Donders G Bellen G Rezeberga D. Aerobic vaginitis in pregnancy. BJOG (2011) 118(10):1163–70. 10.1111/j.1471-0528.2011.03020.x 21668769

[28] Donders G Van Calsteren K Bellen G Reybrouck R Van den Bosch T Riphagen I Predictive value for preterm birth of abnormal vaginal flora, bacterial vaginosis and aerobic vaginitis during the first trimester of pregnancy. BJOG (2009) 116(10):1315–24. 10.1111/j.1471-0528.2009.02237.x 19538417

[29] MacPhee RA Miller WL Gloor GB McCormick JK Hammond J-A Burton JP Influence of the vaginal microbiota on toxic shock syndrome toxin 1 production by *Staphylococcus aureus*. Appl Environ Microbiol (2013) 79(6):1835–42. 10.1128/AEM.02908-12 23315732 PMC3592239

[30] Tansarli GS Kostaras EK Athanasiou S Falagas ME. Prevalence and treatment of aerobic vaginitis among non-pregnant women: evaluation of the evidence for an underestimated clinical entity. Eur J Clin Microbiol Infect Dis (2013) 32(8):977–84. 10.1007/s10096-013-1846-4 23443475

[31] Li J McCormick J Bocking A Reid G. Importance of vaginal microbes in reproductive health. Reprod Sci (2012) 19(3):235–42. 10.1177/1933719111418379 22383775

[32] Donders GG Ruban K Bellen G. Selecting anti-microbial treatment of aerobic vaginitis. Curr Infect Dis Rep (2015) 17(5):24. 10.1007/s11908-015-0477-6 25896749

[33] Donders GG. Definition and classification of abnormal vaginal flora. Best Pract Res Clin Obstet Gynaecol (2007) 21(3):355–73. 10.1016/j.bpobgyn.2007.01.002 17434799

[34] Sørensen UBS Poulsen K Ghezzo C Margarit I Kilian M. Emergence and global dissemination of host-specific *Streptococcus agalactiae* clones. MBio (2010) 1(3):e00178–10. 10.1128/mBio.00178-10 20824105 PMC2932510

[35] Brigtsen AK Dedi L Melby KK Holberg-Petersen M Radtke A Lyng RV Comparison of PCR and serotyping of group B *Streptococcus* in pregnant women: the Oslo GBS-study. J Microbiol Methods (2015) 108:31–5. 10.1016/j.mimet.2014.11.001 25447890

[36] Kwatra G Adrian PV Shiri T Buchmann EJ Cutland CL Madhi SA. Serotype-specific acquisition and loss of group B *Streptococcus* recto-vaginal colonization in late pregnancy. PLoS One (2014) 9(6):e98778. 10.1371/journal.pone.0098778 24979575 PMC4076185

[37] Cools P Jespers V Hardy L Crucitti T Delany-Moretlwe S Mwaura M A multi-country cross-sectional study of vaginal carriage of group B streptococci (GBS) and *Escherichia coli* in resource-poor settings: prevalences and risk factors. PLoS One (2016) 11(1):e0148052. 10.1371/journal.pone.0148052 26811897 PMC4727807

[38] Randis TM Gelber SE Hooven TA Abellar RG Akabas LH Lewis EL Group B *Streptococcus* beta-hemolysin/cytolysin breaches maternal-fetal barriers to cause preterm birth and intrauterine fetal demise in vivo. J Infect Dis (2014) 210(2):265–73. 10.1093/infdis/jiu067 24474814 PMC4092248

[39] Kim S-Y Russell LB Park J Verani JR Madhi SA Cutland CL Cost-effectiveness of a potential group B streptococcal vaccine program for pregnant women in South Africa. Vaccine (2014) 32(17):1954–63. 10.1016/j.vaccine.2014.01.062 24530145

[40] Edwards MS Gonik B. Preventing the broad spectrum of perinatal morbidity and mortality through group B streptococcal vaccination. Vaccine (2013) 31:D66–71. 10.1016/j.vaccine.2012.11.046 23200934

[41] Hill JB Sheffield JS McIntire DD Wendel GD Jr. Acute pyelonephritis in pregnancy. Obstet Gynecol (2005) 105(1):18–23. 10.1097/01.AOG.0000149154.96285.a0 15625136

[42] Wood EG Dillon HC Jr. A prospective study of group B streptococcal bacteriuria in pregnancy. Am J Obstet Gynecol (1981) 140(5):515–20. 10.1016/0002-9378(81)90226-X 7018248

[43] Anderson BL Simhan HN Simons KM Wiesenfeld HC. Untreated asymptomatic group B streptococcal bacteriuria early in pregnancy and chorioamnionitis at delivery. Am J Obstet Gynecol (2007) 196(6):524.e1–5. 10.1016/j.ajog.2007.01.006 17547879

[44] Han C Wu W Fan A Wang Y Zhang H Chu Z Diagnostic and therapeutic advancements for aerobic vaginitis. Arch Gynecol Obstet (2015) 291(2):251–7. 10.1007/s00404-014-3525-9 25367602

[45] Tempera G Furneri PM. Management of aerobic vaginitis. Gynecol Obstet Invest (2010) 70(4):244–9. 10.1159/000314013 21051843

[46] Africa C Kaambo E Stemmet M, editors. Opportunistic vaginal infections implicated in the risk for adverse pregnancy outcomes. 11th International Symposium on Antimicrobial Agents and Resistance (ISAAR) and 3rd International Interscience Conference on Infection and Chemotherapy (ICIC) Vol. 50(Suppl 1), Busan: Int J Antimicrob Agents (2017). p. S84.

